# Upregulated serum granulysin levels in women with antiphospholipid antibody‐associated recurrent miscarriage are downregulated by heparin treatment

**DOI:** 10.1002/rmb2.12460

**Published:** 2022-04-17

**Authors:** Tomoko Ichikawa, Yasuyuki Negishi, Sayuri Kasano, Ryoko Yokote, Mirei Yonezawa, Nozomi Ouchi, Yoshimitsu Kuwabara, Shunji Suzuki, Toshiyuki Takeshita

**Affiliations:** ^1^ 26367 Department of Obstetrics and Gynecology Nippon Medical School Tokyo Japan; ^2^ 26367 Department of Microbiology and Immunology Nippon Medical School Tokyo Japan

**Keywords:** antiphospholipid syndrome, granulysin, heparin, innate immunity, recurrent pregnancy loss

## Abstract

**Purpose:**

Granulysin is a cytotoxic protein that simultaneously activates innate and cellular immunity. The authors aimed to evaluate whether granulysin is associated with the antiphospholipid antibody syndrome and whether heparin changes the granulysin levels.

**Methods:**

A cohort study was performed with women with antiphospholipid antibody‐positive recurrent pregnancy loss (RPL). The authors examined granulysin levels under RPL and evaluated the changes in serum granulysin levels before and 1 week after the commencement of heparin treatment.

**Results:**

Serum granulysin levels before heparin treatment were significantly higher in women who tested positive for one or more types of antiphospholipid antibodies (2.75 ± 1.03 vs. 2.44 ± 0.69, *p* = 0.0341 by Welch's *t* test), particularly anti‐phosphatidylethanolamine antibodies (IgG: 2.98 ± 1.09 vs. 2.51 ± 0.86, *p* = 0.0013; IgM: 2.85 ± 1.09 vs. 2.47 ± 0.77, *p* = 0.0024 by Welch's *t* test). After heparin treatment for 1 week, serum granulysin levels were significantly reduced (*p* = 0.0017 by the paired *t* test). The miscarriage rate was significantly higher in women whose serum granulysin levels were not reduced by heparin treatment (*p* = 0.0086 by Fisher's exact probability test).

**Conclusion:**

The results suggest that heparin may reduce the incidence of miscarriage by suppressing serum granulysin levels.

## Abstract 2

We examined granulysin levels under recurrent pregnancy loss and evaluated the changes in serum granulysin with heparin treatment. The miscarriage rate was significantly higher in women whose serum granulysin levels were not reduced by heparin treatment. The results suggest that heparin may reduce the incidence of miscarriage by suppressing serum granulysin levels.

## 1 INTRODUCTION

Recurrent pregnancy loss (RPL) is a heterogeneous condition with several etiological factors, such as uterine anomalies, prothrombotic disorders, chromosomal anomalies, endocrine dysfunction, and autoimmune and alloimmune disorders. Among these, antiphospholipid antibody syndrome (APS) is recognized as one of the most important causes of RPL. An estimated 20%–40% of women with RPL test positive for antiphospholipid antibodies (aPLs), compared with 2% of women with normal obstetric histories.^1^

Antiphospholipid antibody syndrome is primarily characterized by arterial or venous thrombosis, RPL, fetal growth restriction, and preeclampsia in patients positive for aPLs.^2^ Observations of extensive infarction and thrombosis in the placentas of aPL‐positive women and evidence of systemic thrombosis in patients with APS led to the hypothesis that aPLs cause obstetric complications by inducing thrombosis in the placenta and decidual circulation.^3^ However, not all affected placentas display signs of thrombosis or infarction, and most miscarriages occur before placenta formation, indicating that other mechanisms are responsible for these obstetric complications.

Recent clinical and experimental studies suggest that the pathophysiology of APS‐related pregnancy loss may involve inflammation at the maternal–fetal interface and disruption of normal trophoblast function. Girardi et al.^4^ suggested that activation of the complement cascade is necessary for aPL‐mediated fetal loss. In addition, the presence of neutrophil extracellular traps (NETs) is crucial in APS. In patients with APS, the neutrophils secrete NETs,^5^ and the thrombi formed include those derived from NETs.^6^ It has been postulated that a proportion of RPL cases is attributed to immune reactions.^7^ However, the role of immunity in APS pathogenesis remains unclear.

Heparin in combination with aspirin is currently the standard treatment for pregnant women with APS. The rationale underlying this choice of treatment is based on the anticoagulant properties of heparin because it was originally thought that pregnancy failure associated with aPLs is caused by thrombotic events in the placenta. However, as described above, there may be other mechanisms underlying pregnancy loss, and heparin may be used as an alternative preventive agent. Indeed, heparin has several properties besides its anticoagulant property, including inhibition of aPL binding to β2 glycoprotein I (β2GPI).^8^ Moreover, Girardi et al.^9^ demonstrated that the effectiveness of heparin may be attributed to its complementary inhibitory effects rather than its anticoagulant activity. These observations led us to hypothesize that changes in immunity may contribute to the pathogenesis and clinical manifestations of APS and that heparin treatment may modulate immunity in pregnant women with APS.

We used granulysin, a cytolytic granule protein produced by natural killer (NK) cells,^10^ cytotoxic T lymphocytes (CTLs), natural killer T (NKT) cells, and γδ T cells,^11^ as an indicator of immunity. Granulysin is a demonstrated marker of cell‐mediated immunity,^12^ as well as perforin and granzyme activity.^13^ It is a saposin‐like lipid‐binding protein^14^ that is inserted into cell membranes to induce ion fluxes and apoptosis. Granulysin is composed of two subunits; the 9 kDa subunit exerts cytotoxic effects, whereas the 15 kDa subunit acts as an alarmin,^15^ which stimulates antigen‐presenting cells (APCs) through pattern recognition receptors (PRRs) and is involved in the activation of innate immune cells, including dendritic cells (DCs).^16^ The 15 kDa subunit acts as a damage‐associated molecular pattern and induces preinflammatory reactions.^17^ The 15 kDa subunit also promotes APC maturation^18^, ^19^ and immune cell migration.^20^ Therefore, granulysin can regulate the innate and adaptive arms of the immune system.

Granulysin has tumoricidal and antiviral properties and inhibits the growth of pathogenic bacteria, fungi, and parasites in vitro.^10^, ^21^ It is also a useful biomarker for transplant. In acute graft‐versus‐host disease, serum granulysin levels are markedly increased and correlate with the disease severity.^22^ Granulysin has been detected in the endometrium^4^ and decidua in early pregnancy,^23^ and plasma granulysin concentrations are associated with preeclampsia.^24^ Recently, Nakashima et al.^25^ demonstrated that granulysin produced by uterine NK cells induces apoptosis in extravillous trophoblasts during spontaneous abortion. Tamara et al.^26^ also reported that granulysin causes apoptosis and induces miscarriage. Based on these findings, granulysin can be used for an evaluation of immunity and it may be involved in miscarriage. Moreover, it has been reported that APS may induce miscarriage not only by thrombosis but also by immune response. Here, we investigated the relationship between APS and immunity by examining serum granulysin levels.

## 2 MATERIALS AND METHODS

### 2.1 Patients

**FIGURE 1:**
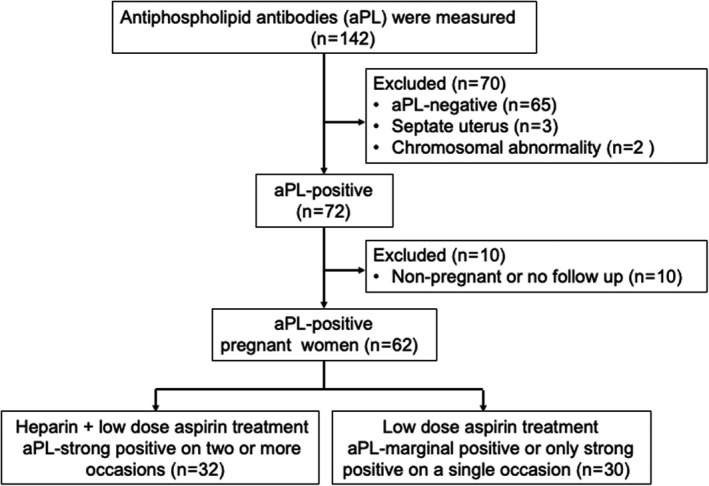
Study flowchart

Women were recruited from the recurrent miscarriage clinic of the Department of Obstetrics and Gynecology at Nippon Medical School Hospital (Tokyo, Japan). A total of 142 women with a history of recurrent miscarriage (two or more consecutive early or late miscarriages) were included in this study. The aPL value was determined for all patients; 72 were found to be positive. Patients negative for aPL (*n* = 65), septate uterus (*n* = 3), and parental chromosomal abnormalities (*n* = 2) were excluded. Non‐pregnant and no follow‐up patients (*n* = 10) were also excluded. A total of 62 aPL‐positive patients became pregnant. Thirty‐two pregnant women whose aPL test was strongly positive (>99th percentile) on two or more occasions, at least 12 weeks apart, were treated with heparin and aspirin (heparin plus aspirin group). Thirty women, whose aPL was marginally positive (≤99th percentile) or strongly positive only on one occasion, received aspirin alone (aspirin‐alone group; Figure 1). Patients with treated and well‐controlled hypothyroidism were not excluded. Patients with protein S deficiency and protein C deficiency were also not excluded because these deficiencies are not completely certified as a cause of RPL as per the internationally accepted criteria. We also included RPL patients with secondary and primary APS; however, steroids or hydroxychloroquine was not used for these patients.

### 2.2 Laboratory assays for aPL detection

The presence or absence of lupus anticoagulant was determined by measuring the activated partial thromboplastin time and diluted Russell viper venom time, and confirmed using mixing studies and platelet neutralization tests.^27^, ^28^ Standard enzyme‐linked immunosorbent assays (ELISAs) against cardiolipin (CL)^29^, ^30^ and phosphatidylethanolamine (PE) were used to detect the presence of IgG and IgM antibodies,^30^ whereas anti‐β2GPI antibodies were used to detect the presence of anti‐CLβ2GPI complex antibody. Values >10 G phospholipids for anti‐CL IgG, >9 M phospholipids for anti‐CL IgM, >3.5 M for anti‐CLβ2GPI complex antibody, >0.3 (95th percentile) for anti‐PE IgG, and >0.45 (95th percentile) for anti‐PE IgM were considered positive.^31^ These tests were performed two times with a 12‐week interval, and if the threshold value was exceeded in both the replicates, the test was considered positive. ELISA was performed by a commercial laboratory (SRL).

### 2.3 Heparin and aspirin treatments

All patients who participated were administered low‐dose aspirin (81 mg/day), 2 weeks before their expected menstrual period, and were instructed to continue treatment in the event of a positive pregnancy test. Heparin treatment began immediately after a positive urinary pregnancy test. The patients self‐administered 5000 U of unfractionated heparin (Caprocin^®^, Sawai Pharmacy) subcutaneously every 12 h.

### 2.4 Blood sample collection

After a positive pregnancy test, blood samples were obtained immediately before and 1 week after the start of treatment in the heparin plus aspirin (*n* = 32) and aspirin‐alone (*n* = 30) groups.

### 2.5 ELISA for detecting serum granulysin

Serum granulysin concentrations were determined by a commercial laboratory (BML). Briefly, microtiter plates (Nunc) were coated with 5 mg/ml anti‐granulysin monoclonal (m)Ab RB1(Mouse IgG1κ) (MBL International Corporation) in 100 mM carbonate buffer and maintained overnight at 4°C. The plates were washed with phosphate‐buffered saline containing 0.1% Tween‐20 (washing buffer) and blocked with 10% fetal bovine serum wash buffer (blocking buffer) for 1–2 h at 37°C. The plates were then sequentially incubated with the following materials at room temperature: samples or standards in blocking buffer for 2 h, 0.1 mg/ml of mouse monoclonal anti‐human granulysin biotinylated RC8 mAb (MBL International Corporation) in blocking buffer for 1 h, and 0.05 U/ml of β‐galactosidase‐conjugated streptavidin (Roche Diagnostics) in washing buffer for 1 h; the plates were washed in washing buffer between each step. After a final wash, the plates were incubated with 0.4 mM 4‐methylumbelliferyl‐β‐D‐galactoside (Sigma‐Aldrich) in 10 mM sodium phosphate buffer (pH 7.0) supplemented with 0.02% bovine serum albumin, 100 mM NaCl, and 1 mM MgCl_2_ at 37°C for 17 h. The reaction was then terminated by adding 100 mM glycine–NaOH (pH 10.3), and the fluorescence intensity was measured using a CytoFluor Series 4000 Multi‐Well Plate Reader (Applied Biosystems) at excitation and emission wavelengths of 360 and 460 nm, respectively.

### 2.6 Detection of NK activity

NK activity was measured against K562 tumor cells at an effector‐to‐target cell ratio of 20:1 using peripheral blood mononuclear cells. This process was done by SRL Laboratory, Tokyo, Japan.

### 2.7 Detection of the Th1/Th2 ratio

Levels of IFN‐γ‐producing Th cells (Th1 cells; CD4+ T lymphocytes with IFN‐γ without IL‐4) and IL‐4‐producing Th cells (Th2 cells; CD4+ T lymphocytes with IL‐4 without IFN‐γ) were analyzed to evaluate the Th1/Th2 ratio by SRL Laboratory, Tokyo, Japan. Laser flow cytometry (Fascinator II; BD Biosciences) was used to measure the levels of Th cells with phorbol 12‐myristate 13‐acetate, lonomycin, Brefeldin A (Sigma‐Aldrich Corp.), CD4 R‐phycoerythrin‐cyanine (PC)‐5 (Immunotec), and fluorescein isothiocyanate (FITC)/IL‐4‐PE (BD Biosciences). After the activated whole blood samples were stained with anti‐CD4‐PC5‐conjugated monoclonal antibodies, lysis of red blood cells and specific intracellular staining were performed according to the manufacturer's instruction using Fast Immune^TM^ IFN‐FITC/IL‐4‐PE (Becton Dickinson Biosciences). The Th1/Th2 cell ratio was calculated using the ratio of IFN‐γ‐positive to IL‐4‐positive cells.

### 2.8 Statistical analysis

Paired data were analyzed using Welch's *t* tests and paired *t* tests (two‐sided). Fisher's exact probability test was used to test pregnancy outcomes. The planned sample size of 30 was based on 80% power, 0.05 significant level, and 0.5 effect size to detect the magnitude of the difference between the two heparin and aspirin treatment groups. All statistical analyses were performed using the JMP 6.0 software (SAS Institute). Differences with *p* values <0.05 were considered significant.

## 3 RESULTS

### 3.1 Patient characteristics

The characteristics of the patients included in this study are outlined in Table 1. The proportion of multiparous women was 21.8%; 6.5% had a history of hypertension disorder of pregnancy, 3.2% had a history of fetal growth restriction, 9.7% had a history of oligohydramnios, and 9.7% had a history of gestational diabetes mellitus. There were no patients with multiple pregnancies or renal disease. In patients with hypertension disorder of pregnancy, when we collected blood sample, since preeclampsia did not occur, the blood pressure was stable. There were 81 patients positive for at least one aPL, which corresponded to 57.0% of the total number of women recruited. The positivity rates were 9.9% for the anti‐CL IgG antibody, 7.8% for the anti‐CL IgM antibody, 24.1% for the anti‐PE IgG antibody, 41.1% for the anti‐PE IgM antibody, 1.4% for the anti‐CLβ2GPI complex, and 2.1% for lupus anticoagulant (LAC). The mean gestational age at initial blood sampling was 5.8 ± 1.3 weeks in the aspirin plus heparin group and 6.0 ± 1.9 weeks in the aspirin group; these results were not significantly different.

**TABLE 1:** Patient characteristics

Characteristics	Value
Age^a^	35.1 ± 4.1 (23–44)
No history of miscarriages^a^	2.68 ± 1.22 (2–7)
No history of early miscarriages (%)	126 (88.7)
No history of late miscarriages (%)	20 (15.6)
No history of parous women (%)	31(21.8)
No history of hypertension disorder of pregnancy	2 (6.5)
No history of fetal growth restriction	1 (3.2)
No history of oligohydramnios	3 (9.7)
No history of gestational diabetes	3 (9.7)
No. of patients with one or more APAs	81 (57.0)
Anti‐CL IgG positive	14 (9.9)
Anti‐CL IgM positive	11 (7.8)
Anti‐PE IgG positive	34 (24.1)
Anti‐PE IgM positive	58 (41.1)
Anti‐CLβ2GPI complex antibody positive	2 (1.4)
LAC positive	3 (2.1)

Abbreviations: anti‐CL IgG, anti‐cardiolipin IgG; anti‐CL IgM, anti‐cardiolipin IgM; anti‐CLβ2GPI complex antibody, anti‐cardiolipin β2GPI complex antibody; anti‐PE IgG, anti‐phosphatidylethanolamine IgG; anti‐PE IgM, anti‐phosphatidylethanolamine IgM; APAs, antiphospholipid antibodies.

^a^ Values are presented as the mean ± SD (range).

### 3.2 Serum granulysin levels in women with and without aPLs

Serum granulysin concentrations were significantly higher in women who tested positive for one or more aPLs than in women who tested negative (2.75 ± 1.03 ng/ml vs. 2.44 ± 0.69 ng/ml; *p* = 0.0341). There were no significant differences in the granulysin concentrations of women with and without anti‐CL IgG or IgM antibodies; however, the concentrations were significantly higher in women who tested positive for either anti‐PE IgG or IgM antibodies (Table 2). No correlation was observed between the levels of granulysin and anti‐CL IgG/IgM and anti‐PE IgG/IgM (*r* = −0.1256 for anti‐CL IgG, 95% CI = −0.307–0.063; *r* = −0.1543 for anti‐CL IgM, 95% CI = −0.346–0.049; *r* = 0.1137 for anti‐PE IgG, 95% CI −0.064–0.285; and *r* = −0.0828 for anti‐PE IgM, 95% CI = −0.1439–0.203) as shown in Figure S3.

**TABLE 2:** Serum granulysin concentration in women with or without antiphospholipid antibodies

	Positive (*n*)	Negative (*n*)	*p* value
Antiphospholipid antibodies	2.75 ± 1.03 (81)	2.44 ± 0.69 (60)	0.0341^a^
Anti‐CL IgG	2.56 ± 1.40 (14)	2.62 ± 0.85 (127)	0.879^a^
Anti‐CL IgM	2.39 ± 1.04 (11)	2.63 ± 0.90 (130)	0.390^b^
Anti‐PE IgG	2.98 ± 1.09 (34)	2.51 ± 0.86 (107)	0.013^b^
Anti‐PE IgM	2.85 ± 1.09 (58)	2.47 ± 0.77 (83)	0.024^a^

Abbreviations: anti‐CL IgG, anti‐cardiolipin IgG; anti‐CL IgM, anti‐cardiolipin IgM; anti‐PE IgG, anti‐phosphatidylethanolamine IgG; anti‐PE IgM, anti‐phosphatidylethanolamine IgM.

^a^ Welch's *t* test.

^b^ Student's *t* test.

### 3.3 Characteristics of patients treated with aspirin or heparin plus aspirin

Thirty‐two women received aspirin plus heparin, and thirty received aspirin alone. The mean ages of women in the aspirin plus heparin and aspirin‐alone groups were 35.5 ± 3.84 (range: 30–41) years and 33.2 ± 2.94 (range: 29–38) years, respectively, which were not significantly different (Table 3).

**TABLE 3:** Characteristics of patients treated with heparin and aspirin or aspirin alone

	Heparin + Aspirin	Aspirin	
No. of patients	32	30	
Age^a^	35.5 ± 3.84 (30–41)	33.2 ± 2.94 (29–38)	ns
No. of miscarriages^a^	2.87 ± 1.41 (2–6)	2.47 ± 0.92 (2–5)	ns

Abbreviation: ns, not significant.

^a^ Values are presented as the mean ± SD (range).

### 3.4 Changes in serum granulysin concentration after heparin treatment

**FIGURE 2:**
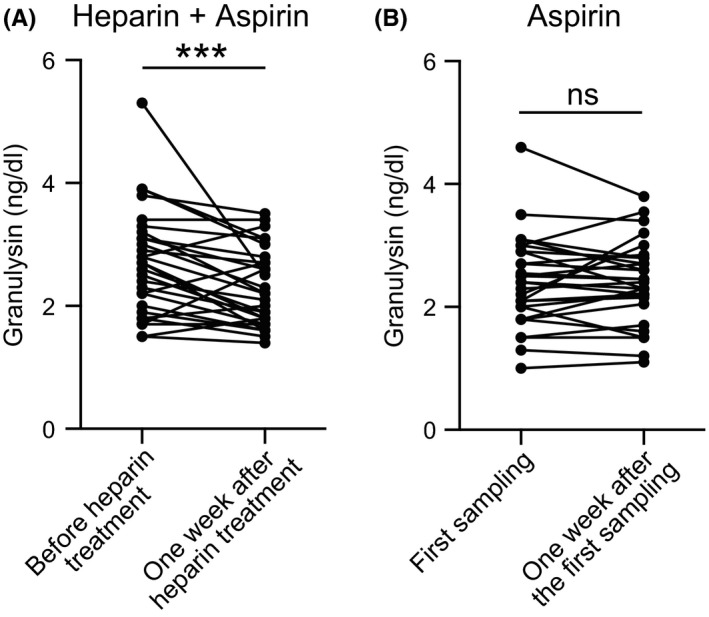
Changes in serum granulysin levels in patients with RPL treated with heparin and aspirin. Serum granulysin concentration was assayed using ELISA. (a) Serum granulysin levels of patients treated with heparin plus aspirin combination therapy were measured before and 1 week after the initiation of heparin treatment (*n* = 32). (b) Serum granulysin levels of patients treated with aspirin alone (*n* = 30). The gestational age of sampling is the same as in the heparin plus aspirin combination therapy group. ***p < 0.001: paired *t* test

**FIGURE 3:**
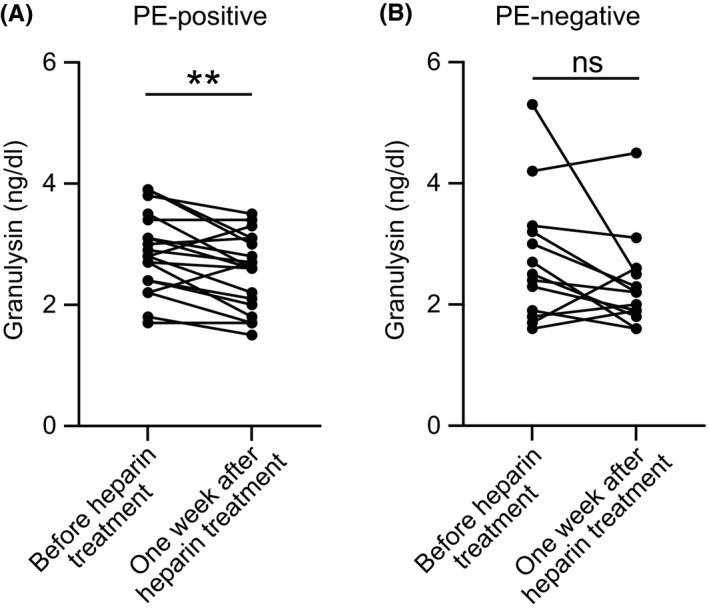
Changes in serum granulysin levels in patients with PE‐positive and PE‐negative RPL. Serum granulysin concentration was assayed using ELISA. (a, b) Serum granulysin levels of PE‐positive (a) and PE‐negative (b) patients treated with heparin plus aspirin were measured before and 1 week after the initiation of heparin treatment (*n* = 19 and 13, respectively). (The gestational age of sampling is the same as in the heparin plus aspirin combination therapy group.) **p < 0.01: paired *t* test

The mean serum granulysin level before heparin treatment was 2.77 ± 0.82 ng/ml, which significantly decreased to 2.37 ± 0.67 ng/ml (*p* = 0.0007) 1 week after the initiation of heparin treatment (Figure 2a). Serum granulysin levels in the aspirin group did not differ significantly during this period (*p* = 0.84; Figure 2b). In addition, the change in granulysin levels before and 1 week after heparin treatment showed a significant difference in PE‐positive patients (2.85 ± 0.15 ng/dl vs. 2.55 ± 0.14 ng/dl, *p* = 0.0047; Figure 3a) but not in PE‐negative patients (2.76 ± 0.30 ng/dl vs. 2.32 ± 0.22 ng/dl, *p* = 0.107; Figure 3b). Moreover, there was no significant difference in granulysin levels between PE‐positive and PE‐negative patients 1 week after heparin and aspirin treatment (2.60 ± 0.14 ng/dl vs. 2.00 ± 0.19 ng/dl, *p* = 0.337; data not shown). No changes in NK cell activity or type 1/2 helper T‐cell (Th1/Th2) ratio were observed after heparin treatment (*p* = 0.20 and 0.82, respectively; Figures S1 and S2).

### 3.5 Relationship between pregnancy outcomes and changes in serum granulysin levels

**FIGURE 4:**
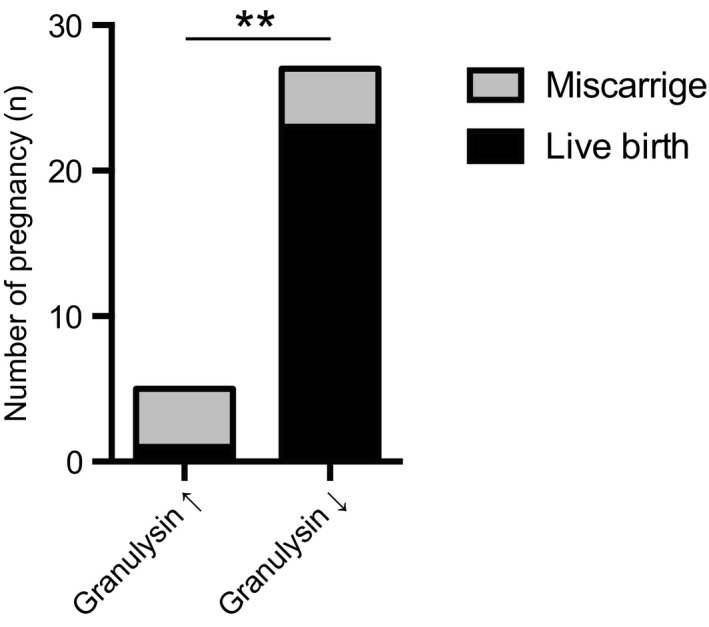
Pregnancy outcome stratified by granulysin levels after heparin plus aspirin treatment. **p < 0.05: Fisher's exact probability test

Of the 32 patients treated with heparin plus aspirin, 27 showed decreased serum granulysin levels and 5 showed elevated levels. Miscarriages occurred in 80% (4/5) of patients with increased granulysin levels and in 15% (4/27) of those with decreased granulysin levels. Therefore, when heparin treatment resulted in decreased serum granulysin levels, a significantly lower miscarriage rate was observed compared with that in the cases in which heparin treatment resulted in increased serum granulysin levels (*p* = 0.0086; Figure 4).

## 4 DISCUSSION

In this study, we found that serum granulysin levels were higher in women positive for aPLs, especially anti‐PE antibodies, and that heparin treatment significantly decreased the serum granulysin levels. The miscarriage rate was significantly lower in women whose serum granulysin levels were reduced by heparin treatment, indicating that heparin can reduce granulysin levels to prevent miscarriage. The mechanism by which heparin regulates granulysin levels remains unknown.

Although heparin primarily exerts antithrombotic effects,^32^, ^33^ it is also reported to attenuate apoptosis in the placenta and villi^34^, ^35^ and has a suppressive effect on complement activation.^9^

In this study, we analyzed the NK cell activity and the Th1/Th2 ratio in the peripheral blood of patients. It is unclear why granulysin levels were not correlated with the NK cell activity. In general, granulysin is produced by CTLs and by NK and NKT cells. Indeed, the production of cytotoxic factors (perforin and granzyme) by uterine NKT cells was significantly higher than that by uterine NK cells in our previous study.^36^ Therefore, NK cell activation may not have decreased in response to heparin treatment because granulysin was produced by NK cells and by CTLs and NKT cells. It is also unclear why granulysin levels were not correlated with the Th1/Th2 ratio in the present study. The Th1/Th2 ratio is used to evaluate interferon‐gamma (IFN‐γ) (corresponding to Th1) and interleukin (IL)‐4 (corresponding to Th2) levels. However, in addition to IFN‐γ, other cytokines, such as IL‐12, tumor necrosis factor‐α, and IL‐2, are involved in the Th1 response.^37^, ^38^ Because Th2 cytokines are also associated with the expression of IL‐10 and IL‐13 in addition to that of IL‐4, their levels cannot be simply evaluated by the Th1/Th2 ratio. Moreover, because Th17 and regulatory T cells also share a complex association,^39^ it is necessary to investigate the correlation between various cytokines and granulysin in future studies. In a previous study, we observed elevated levels of cytotoxic granules and inflammatory cytokines, such as IL‐2 and IL‐12, as well as of perforin and granzyme B, at the implantation site rather than at the systemic level in a murine miscarriage model induced by α‐galactosylceramide (α‐GalCer).^40^ Nakashima et al.^25^ also reported that granulysin produced by uterine NK cells induces the apoptosis of extravillous trophoblasts, and 85% of NK cells in the decidua of the uterus contain granulysin, which is twice the proportion observed in the periphery.^41^ In the future, it will be necessary to investigate local changes in these cells in aPL‐positive patients.

We previously reported that activated DCs take up α‐GalCer and activate NKT cells via CD1d and IL‐12, which results in a miscarriage.^40^ In this study, the abundance of uterine NKT cells was observed to increase after miscarriage. These cells express multiple cytokines, such as IL‐2 and IFN‐γ, along with perforin and granzyme, at high levels. However, the actual effector has not been identified. Moreover, NKT cells produce granulysin,^11^ which has been reported to induce miscarriage.^26^ Therefore, α‐GalCer‐induced sterile inflammatory miscarriage may be associated with granulysin expression. The alarmin activity of granulysin^15^ may also play a role in this. Recent studies have revealed that granulysin also acts as an alarmin leading to the activation of TLR4 on APCs.^20^ Alarmins stimulate APCs, and granulysin, which is present at high levels in patients with APS (especially in those who test positive for anti‐PE antibodies), activates PRR‐expressing DCs, which may further enhance granulysin secretion by NKT cells, NK cells, and CTLs, and eventually induce miscarriage via its direct effect on apoptosis.

HMGB1, an alarmin that is secreted by necrotic cells or is passively secreted by immune cells into the extracellular matrix, activates APCs, including DCs, as well as granulysin.^42^ The expression of HMGB1 is increased in patients with APS^43^ and in individuals with RPL of unknown etiology.^44^ These findings and our results indicate that granulysin, as an alarmin, may also be involved in the induction of miscarriage.

In an earlier study, patients with APS who tested positive for anti‐PE antibodies showed high granulysin levels, and when this persisted for more than 12 weeks, the miscarriage rate was found to be high.^45^ PE is an anti‐inflammatory lipid^46^; therefore, anti‐PE antibody‐positive patients may show higher levels of inflammation, increased immune activity (including granulysin production), and a higher risk of miscarriage. The mechanism by which heparin reduces granulysin levels in anti‐PE antibody‐positive patients is unclear. It was speculated that granulysin has an affinity for the phospholipid phosphatidylserine (PS), and PS is converted to PE in the base exchange reaction. Therefore, granulysin may have been specifically elevated by the anti‐PE antibody. In addition, PS is normally retained in the cytoplasm, but is exposed to the cell surface when apoptosis occurs in the cells. Therefore, it is possible that heparin regulates villous cell apoptosis, resulting in a decrease in the levels of PS, PE, and granulysin. In our study, when heparin treatment reduced the serum granulysin levels, the miscarriage rate was found to be lower than when the levels increased. Therefore, serum granulysin concentration may be useful as a clinical marker of RPL. In addition, the prophylactic administration of heparin before pregnancy to women with high granulysin levels may be effective. Further research will be required to investigate this.

Indeed, anti‐PE antibodies are not included in the diagnostic criteria for APS but for seronegative APS. This indicates that granulysin may have negative effects on seronegative APS. Moreover, granulysin levels might be elevated by infections and inflammation regardless of aPL. In future studies, we intend to evaluate the effects of granulysin on seronegative APS, for which no treatment has been established, and would test the effectiveness of heparin for seronegative APS.

Preimplantation genetic testing for aneuploidy is increasingly being performed on patients with RPL, which has helped reduce the proportion of miscarriages of unknown etiologies. However, cases of recurrent miscarriage of unknown etiology continue to be reported, including some cases caused by immunological abnormalities. We have already reported the involvement of anti‐C9 antibodies in patients with recurrent miscarriage of unknown etiology.^47^ In the future, we intend to increase the number of recorded granulysin measurements, establish key clinical thresholds, appropriately identify patients who require heparin treatment, and commence treatment to reduce the risk of miscarriage. A limitation of this study is its relatively small sample size, which precluded the investigation of anti‐β2GPI antibodies or other APS‐related antibodies. We intend to increase the sample size in future studies and examine the association between serum granulysin levels and multiple antibodies. Serum granulysin levels upregulated in aPL‐positive patients were downregulated in response to heparin treatment. This suggests that aPLs are associated with the immune system, and heparin plays a role in inhibiting the immune response. Based on these findings, it may be possible to measure granulysin levels in aPL‐positive RPL patients of unknown cause and to avoid miscarriage with heparin treatment, if the values are high.

## CONFLICT OF INTEREST

The authors declare no conflict of interest.

## HUMAN RIGHTS STATEMENTS AND INFORMED CONSENT

All experiments were in accordance with the ethical standards of the responsible committee on human experimentation and with the Helsinki Declaration of 1964 and its later amendments. This study involved the analysis of human blood samples. Written informed consent was obtained from all study participants.

## APPROVAL BY ETHICS COMMITTEE

The Committee of Nippon Medical School Hospital approved the collection and use of biological materials for this study, and all experiments were performed according to the guidelines (19–03–56).

## Supporting information

Fig S1‐S3

## References

[1] Di Simone N , Luigi MP , Marco D , et al. Pregnancies complicated with antiphospholipid syndrome: the pathogenic mechanism of antiphospholipid antibodies: a review of the literature. Ann N Y Acad Sci. 2007;1108:505‐514. 17894016 10.1196/annals.1422.054

[2] Greaves M . Antiphospholipid syndrome: state of the art with emphasis on laboratory evaluation. Haemostasis. 2000;30(Suppl 2):16‐25. 11251337 10.1159/000054159

[3] De Wolf F , Carreras LO , Moerman P , Vermylen J , Van Assche A , Renaer M . Decidual vasculopathy and extensive placental infarction in a patient with repeated thromboembolic accidents, recurrent fetal loss, and a lupus anticoagulant. Am J Obstet Gynecol. 1982;142(7):829‐834. 6801984 10.1016/s0002-9378(16)32527-3

[4] Girardi G , Berman J , Redecha P , et al. Complement C5a receptors and neutrophils mediate fetal injury in the antiphospholipid syndrome. J Clin Invest. 2003;112(11):1644‐1654. 14660741 10.1172/JCI18817 PMC281643

[5] Yalavarthi S , Gould TJ , Rao AN , et al. Release of neutrophil extracellular traps by neutrophils stimulated with antiphospholipid antibodies: a newly identified mechanism of thrombosis in the antiphospholipid syndrome. Arthritis Rheumatol. 2015;67(11):2990‐3003. 26097119 10.1002/art.39247 PMC4626310

[6] Meng H , Yalavarthi S , Kanthi Y , et al. In vivo role of neutrophil extracellular traps in antiphospholipid antibody‐mediated venous thrombosis. Arthritis Rheumatol. 2017;69(3):655‐667. 27696751 10.1002/art.39938 PMC5329054

[7] Laird SM , Tuckerman EM , Cork BA , Linjawi S , Blakemore AI , Li TC . A review of immune cells and molecules in women with recurrent miscarriage. Hum Reprod Update. 2003;9(2):163‐174. 12751778 10.1093/humupd/dmg013

[8] Guerin J , Sheng Y , Reddel S , Iverson GM , Chapman MG , Krilis SA . Heparin inhibits the binding of beta 2‐glycoprotein I to phospholipids and promotes the plasmin‐mediated inactivation of this blood protein. Elucidation of the consequences of the two biological events in patients with the anti‐phospholipid syndrome. J Biol Chem. 2002;277(4):2644‐2649. 11711550 10.1074/jbc.M110176200

[9] Girardi G , Redecha P , Salmon JE . Heparin prevents antiphospholipid antibody‐induced fetal loss by inhibiting complement activation. Nat Med. 2004;10(11):1222‐1226. 15489858 10.1038/nm1121

[10] Krensky AM , Clayberger C . Granulysin: a novel host defense molecule. Am J Transplant. 2005;5(8):1789‐1792. 15996224 10.1111/j.1600-6143.2005.00970.x

[11] Gansert JL , Kiessler V , Engele M , et al. Human NKT cells express granulysin and exhibit antimycobacterial activity. J Immunol. 2003;170(6):3154‐3161. 12626573 10.4049/jimmunol.170.6.3154

[12] Ogawa K , Takamori Y , Suzuki K , et al. Granulysin in human serum as a marker of cell‐mediated immunity. Eur J Immunol. 2003;33(7):1925‐1933. 12884856 10.1002/eji.200323977

[13] Clayberger C , Krensky AM . Granulysin. Curr Opin Immunol. 2003;15(5):560‐565. 14499265 10.1016/s0952-7915(03)00097-9

[14] Pena SV , Krensky AM . Granulysin, a new human cytolytic granule‐associated protein with possible involvement in cell‐mediated cytotoxicity. Semin Immunol. 1997;9(2):117‐125. 9194222 10.1006/smim.1997.0061

[15] Sparrow E , Bodman‐Smith MD . Granulysin: the attractive side of a natural born killer. Immunol Lett. 2020;217:126‐132. 31726187 10.1016/j.imlet.2019.11.005

[16] Roh JS , Sohn DH . Damage‐associated molecular patterns in inflammatory diseases. Immune Netw. 2018;18(4):e27. 30181915 10.4110/in.2018.18.e27 PMC6117512

[17] Cai J , Wen J , Bauer E , Zhong H , Yuan H , Chen AF . The role of HMGB1 in cardiovascular biology: danger signals. Antioxid Redox Signal. 2015;23(17):1351‐1369. 26066838 10.1089/ars.2015.6408

[18] Clayberger C , Finn MW , Wang T , et al. 15 kDa granulysin causes differentiation of monocytes to dendritic cells but lacks cytotoxic activity. J Immunol. 2012;188(12):6119‐6126. 22586033 10.4049/jimmunol.1200570 PMC3370151

[19] Castiello L , Stroncek DF , Finn MW , et al. 15 kDa Granulysin versus GM‐CSF for monocytes differentiation: analogies and differences at the transcriptome level. J Transl Med. 2011;9:41. 21501511 10.1186/1479-5876-9-41 PMC3094223

[20] Tewary P , Yang D , de la Rosa G , et al. Granulysin activates antigen‐presenting cells through TLR4 and acts as an immune alarmin. Blood. 2010;116(18):3465‐3474. 20660289 10.1182/blood-2010-03-273953 PMC2981473

[21] Stenger S , Hanson DA , Teitelbaum R , et al. An antimicrobial activity of cytolytic T cells mediated by granulysin. Science. 1998;282(5386):121‐125. 9756476 10.1126/science.282.5386.121

[22] Nagasawa M , Ogawa K , Imashuku S , Mizutani S . Serum granulysin is elevated in patients with hemophagocytic lymphohistiocytosis. Int J Hematol. 2007;86(5):470‐473. 18192122 10.1007/BF02984011

[23] Mincheva‐Nilsson L , Nagaeva O , Sundqvist KG , Hammarstrom ML , Hammarstrom S , Baranov V . gammadelta T cells of human early pregnancy decidua: evidence for cytotoxic potency. Int Immunol. 2000;12(5):585‐596. 10784604 10.1093/intimm/12.5.585

[24] Sakai M , Ogawa K , Shiozaki A , et al. Serum granulysin is a marker for Th1 type immunity in pre‐eclampsia. Clin Exp Immunol. 2004;136(1):114‐119. 15030522 10.1111/j.1365-2249.2004.02414.x PMC1808986

[25] Nakashima A , Shiozaki A , Myojo S , et al. Granulysin produced by uterine natural killer cells induces apoptosis of extravillous trophoblasts in spontaneous abortion. Am J Pathol. 2008;173(3):653‐664. 18688023 10.2353/ajpath.2008.071169 PMC2527087

[26] Gulic T , Laskarin G , Dominovic M , et al. Granulysin‐mediated apoptosis of trophoblasts in blighted ovum and missed abortion. Am J Reprod Immunol. 2018;80(3):e12978. 29774968 10.1111/aji.12978

[27] Fujioka T . Introducing the Hemos Iel Thrombophilia Screening Panel. Igaku to Yakugaku. 2016;73:621‐627.

[28] Pengo V , Tripodi A , Reber G , et al. Update of the guidelines for lupus anticoagulant detection. Subcommittee on Lupus Anticoagulant/Antiphospholipid Antibody of the Scientific and Standardisation Committee of the International Society on Thrombosis and Haemostasis. J Thromb Haemost. 2009;7(10):1737‐1740. 19624461 10.1111/j.1538-7836.2009.03555.x

[29] Okada J . Usefulness of the MESACUP cardiolipin test. Igaku to Yakugaku. 1996;36:1389‐1394.

[30] Kaburagi J . Measuring IgM anti‐cardiolipin antibody Development of enzyme‐linked immunosorbent assay (ELISA) and its clinical usefulness. Igaku to Yakugaku. 2000;43:1183‐1188.

[31] Sugi T , Katsunuma J , Izumi S , McIntyre JA , Makino T . Prevalence and heterogeneity of antiphosphatidylethanolamine antibodies in patients with recurrent early pregnancy losses. Fertil Steril. 1999;71(6):1060‐1065. 10360910 10.1016/s0015-0282(99)00119-3

[32] Bates SM , Rajasekhar A , Middeldorp S , et al. American Society of Hematology 2018 guidelines for management of venous thromboembolism: venous thromboembolism in the context of pregnancy. Blood Adv. 2018;2(22):3317‐3359. 30482767 10.1182/bloodadvances.2018024802 PMC6258928

[33] Bates SM , Greer IA , Middeldorp S , Veenstra DL , Prabulos AM , Vandvik PO . VTE, thrombophilia, antithrombotic therapy, and pregnancy: Antithrombotic Therapy and Prevention of Thrombosis, 9th ed: American College of Chest Physicians Evidence‐Based Clinical Practice Guidelines. Chest. 2012;141(2 Suppl):e691S‐e736S. 22315276 10.1378/chest.11-2300 PMC3278054

[34] Bose P , Black S , Kadyrov M , et al. Heparin and aspirin attenuate placental apoptosis in vitro: implications for early pregnancy failure. Am J Obstet Gynecol. 2005;192(1):23‐30. 15671997 10.1016/j.ajog.2004.09.029

[35] Hills FA , Abrahams VM , González‐Timón B , et al. Heparin prevents programmed cell death in human trophoblast. Mol Hum Reprod. 2006;12(4):237‐243. 16556679 10.1093/molehr/gal026

[36] Negishi Y , Ichikawa T , Takeshita T , Takahashi H . Miscarriage induced by adoptive transfer of dendritic cells and invariant natural killer T cells into mice. Eur J Immunol. 2018;48(6):937‐949. 29520760 10.1002/eji.201747162

[37] Romagnani S . Th1/Th2 cells. Inflamm Bowel Dis. 1999;5(4):285‐294. 10579123 10.1097/00054725-199911000-00009

[38] Romagnani S . T‐cell subsets (Th1 versus Th2). Ann Allergy Asthma Immunol. 2000;85(1):9‐18; quiz 18, 21. 10923599 10.1016/S1081-1206(10)62426-X

[39] Morita K , Tsuda S , Kobayashi E , et al. Analysis of TCR repertoire and PD‐1 expression in decidual and peripheral CD8. Front Immunol. 2020;11:1082. 32582176 10.3389/fimmu.2020.01082 PMC7283903

[40] Ichikawa T , Negishi Y , Shimizu M , Takeshita T , Takahashi H . α‐Galactosylceramide‐activated murine NK1.1(+) invariant‐NKT cells in the myometrium induce miscarriages in mice. Eur J Immunol. 2016;46(8):1867‐1877. 27198610 10.1002/eji.201545923 PMC5089647

[41] Vujaklija DV , Gulic T , Sucic S , et al. First trimester pregnancy decidual natural killer cells contain and spontaneously release high quantities of granulysin. Am J Reprod Immunol. 2011;66(5):363‐372. 21623991 10.1111/j.1600-0897.2011.01015.x

[42] van Beijnum JR , Buurman WA , Griffioen AW . Convergence and amplification of toll‐like receptor (TLR) and receptor for advanced glycation end products (RAGE) signaling pathways via high mobility group B1 (HMGB1). Angiogenesis. 2008;11(1):91‐99. 18264787 10.1007/s10456-008-9093-5

[43] Manganelli V , Truglia S , Capozzi A , et al. Alarmin HMGB1 and soluble RAGE as new tools to evaluate the risk stratification in patients with the antiphospholipid syndrome. Front Immunol. 2019;10:460. 30923525 10.3389/fimmu.2019.00460 PMC6426766

[44] Zou H , Yin J , Zhang Z , et al. Destruction in maternal‐fetal interface of URSA patients via the increase of the HMGB1‐RAGE/TLR2/TLR4‐NF‐κB signaling pathway. Life Sci. 2020;250:117543. 32169518 10.1016/j.lfs.2020.117543

[45] Yonezawa M , Kuwabara Y , Ono S , Ouchi N , Ichikawa T , Takeshita T . Significance of anti‐phosphatidylethanolamine antibodies in the pathogenesis of recurrent pregnancy loss. Reprod Sci. 2020;27(10):1888‐1893. 32548803 10.1007/s43032-020-00208-4

[46] Ireland R , Schwarz B , Nardone G , et al. Unique francisella phosphatidylethanolamine acts as a potent anti‐inflammatory lipid. J Innate Immun. 2018;10(4):291‐305. 29969788 10.1159/000489504 PMC6757151

[47] Kuwabara Y , Katayama A , Kurihara S , Orimo H , Takeshita T . Immunoproteomic identification of anti‐C9 autoimmune antibody in patients with seronegative obstetric antiphospholipid syndrome. PLoS One. 2018;13(6):e0198472. 29894483 10.1371/journal.pone.0198472 PMC5997311

