# Supplementary material for: Upregulated serum granulysin levels in women with antiphospholipid antibody‐associated recurrent miscarriage are downregulated by heparin treatment
Source: Reprod Med Biol. 2022 Apr 17;21(1):e12460. doi: 10.1002/rmb2.12460 (PMC9013493; doi:10.1002/rmb2.12460)
Supplement: Supplementary file 1 — Fig S1‐S3 [file RMB2-21-e12460-s001.pdf]

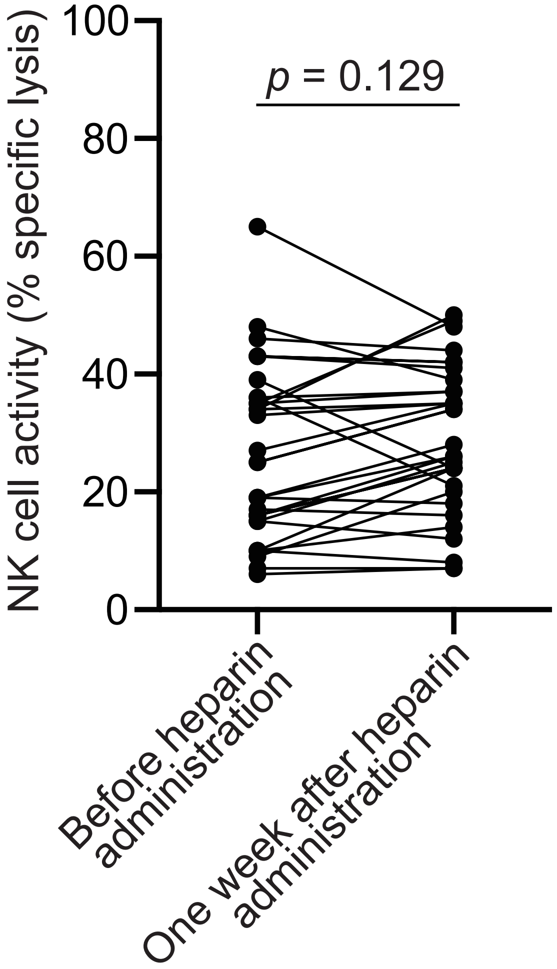

Supplementary Information 1. Changes of NK cell activity in peripheral blood for the patients treated with combination therapy of aspirin and heparin (n = 32). The left plots show the values before heparin administration, and the right plots show the values one week after heparin administration. Peripheral blood mononuclear cells were isolated and incubated with 51Cr-labeled K562 target cells at an E/T ratio of 20:1. Percent specific lysis was calculated. Paired t-test

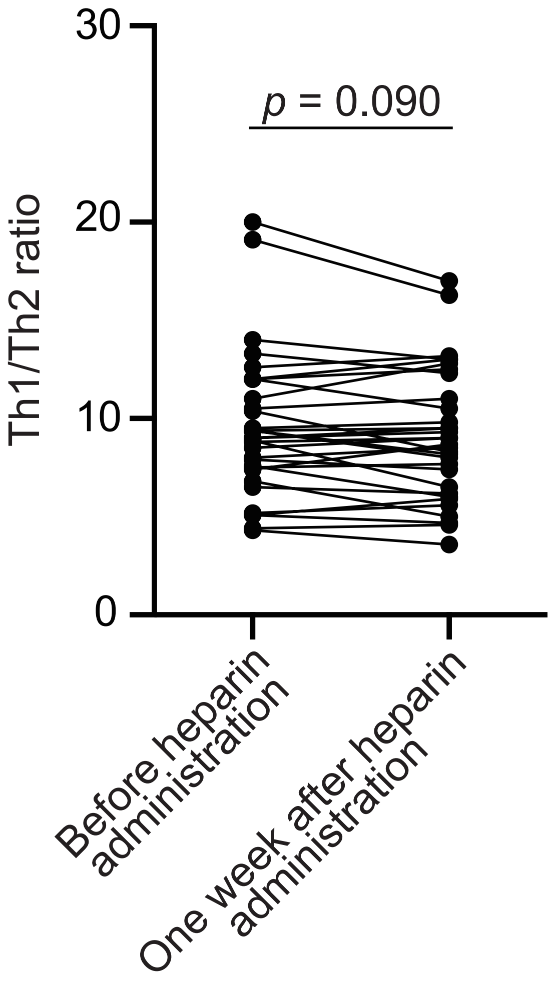

Supplementary Information 2. Changes of Th1/Th2 ratio in peripheral blood for the patients treated with combination therapy of aspirin and heparin (n = 32). The left plots show the values before heparin administration, and the right plots show the values one week after heparin administration. Peripheral blood mononuclear cells were isolated and stimulated with PMA; CD4+ IFN-γ+ IL-4– cells were identified as Th1 cells, whereas CD4+ IFN-γ- IL-4+ cells were identified as Th2 cells. The Th1/Th2 cell ratios were calculated. Paired t-test

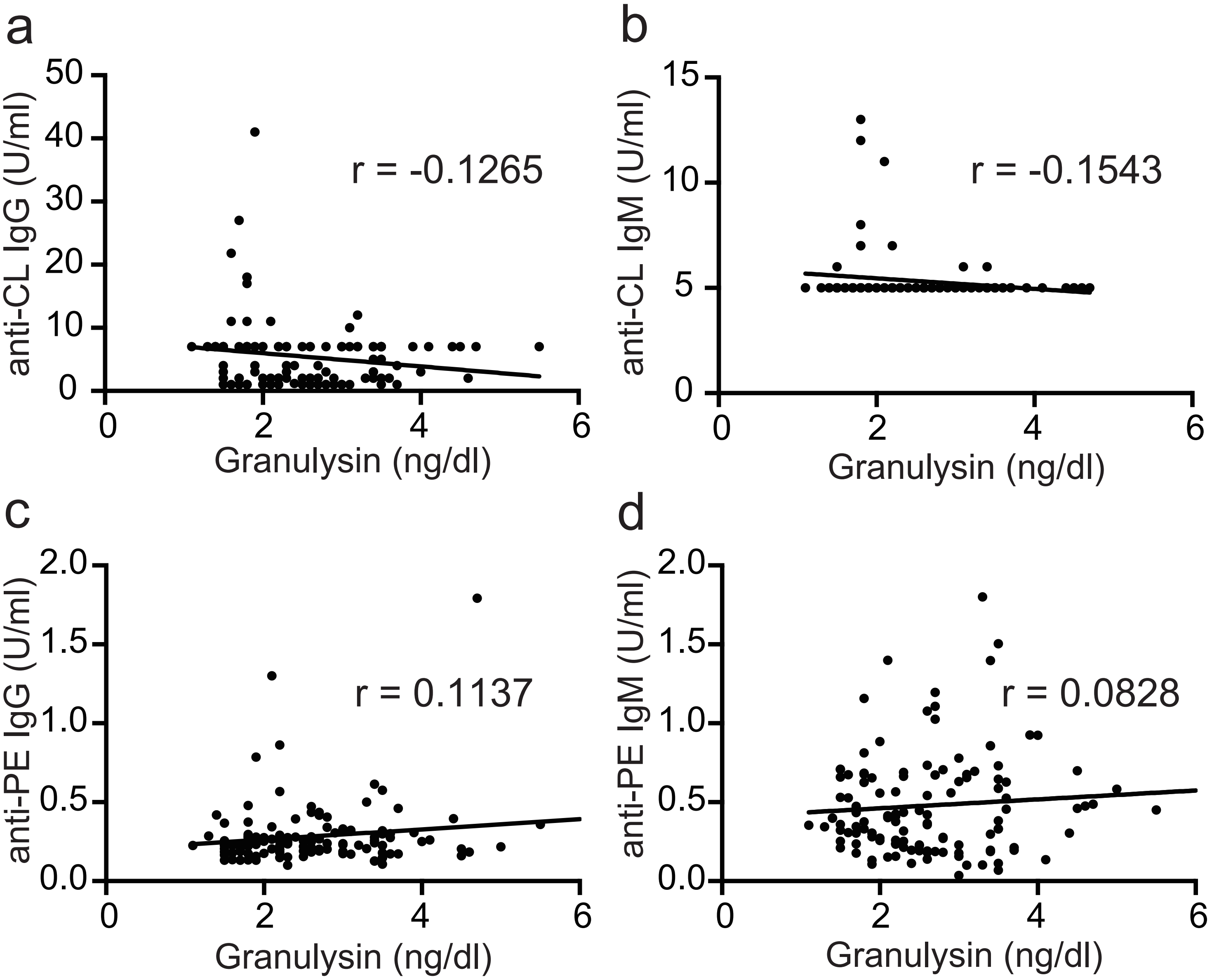

Supplementary Information 3. Correlation between the values of granulysin and the values of anti-CL IgG (a) / IgM (b) and anti-PE IgG (c) / IgM (d). Spearman's rank correlation coefficient
